# Commentary: Clinical Correlates of Raphe Serotonergic Dysfunction in Early Parkinson’s Disease

**DOI:** 10.3389/fneur.2015.00261

**Published:** 2015-12-21

**Authors:** Claudio Liguori, Mariangela Pierantozzi, Enrica Olivola, Nicola B. Mercuri, Alessandro Stefani

**Affiliations:** ^1^Neurophysiopathology Unit, Department of Systems Medicine, University of Rome “Tor Vergata”, Rome, Italy; ^2^Department of Systems Medicine, Movement Disorders Centre, Fondazione Policlinico Tor Vergata, Rome, Italy; ^3^IRCCS Fondazione Santa Lucia, Rome, Italy

**Keywords:** CSF, neuroimaging, SPECT, serotonin, 5-hydroxyindolacetic acid

Growing and clinical evidence supports the conclusion that Parkinson’s Disease (PD) is a complex multisystem disorder not exclusively affecting the dopaminergic circuits. In fact, the dopaminergic neuronal loss does not cover all the clinical aspects of PD. Therefore, different non-dopaminergic neurotransmitter systems have been invoked as playing a role in the PD clinical picture. Principally, the inefficiency of serotonergic circuitry has been demonstrated in PD animal models, as well as in *post-mortem* and *in vivo* human studies.

In the recent article published in *Brain*, Zahi Qamhawi and colleagues interrogated ^123^I-FP-CIT single-photon emission computed tomography documenting raphe serotonergic dysfunction in a large group of early PD patients compared to a subset of possible PD patients without evidence of dopaminergic deficit (SWEDD) and a population of healthy controls (1). This paper combined an accurate clinical evaluation with a sophisticated neuroimaging protocol and documented in PD patients a mean raphe serotonin (5-HT) transporter availability significantly lower than both SWEDD patients and controls. These findings, achieved in a large cohort of PD patients, enforced previous autoptic examinations documenting serotonergic neurons loss due to Lewy body pathology in the raphe nuclei of PD patients (2). Accordingly, neuroimaging studies have shown the progressive 5-HT transporter availability reduction in the raphe nuclei as PD pathology progresses (3–5).

It is well accepted that concentration of 5-HT and its metabolite 5-hydroxyindolacetic acid (5-HIAA) in CSF reflects the serotonergic metabolism and turnover in the CNS (6, 7). We performed a case-control study investigating CSF levels of 5-HT and 5-HIAA in a cohort of PD patients, after a 3-day dopaminergic therapy withdrawal and in absence of serotonergic agents (8). We demonstrated the significant reduction of CSF 5-HT and 5-HIAA concentrations in PD patients compared to controls. We also found the significant reduction of CSF 5-HT and 5-HIAA in the PD population with respect to Alzheimer’s Disease patients (8), thus highlighting that the impairment of serotonergic system could represent a specific effect of synuclein-mediated neurodegeneration (8). As a matter of fact, novel experimental studies in alpha-synuclein (α-syn) mouse model of PD showed the impairment of serotonergic system owing to the intracellular accumulation of α-syn in serotonergic neurons coupled with the reduction of 5-HT levels in lower brainstem (9).

Based on the clinical presentation, Qamhawi and collegues divided PD patients into two subgroups corresponding to patients with and without resting tremor. Remarkably, patients with tremor had lower mean raphe 5-HT transporter availability than patients unaffected by tremor. Since tremor amplitude, constancy, and severity negatively correlated with raphe 5-HT transporter availability in the whole PD cohort, authors suggested that the serotonergic system inefficiency could be responsible for parkinsonian tremor. This finding was consistent with previous studies detecting an association between 5-HT receptor availability in the raphe nuclei and severity of parkinsonian tremor (10). However, in our CSF study, we found no differences in CSF 5-HT and 5-HIAA levels between tremor dominant and non-tremor dominant patients. This discrepancy could be ascribed to the fact that Qamhawi and collegues studied *de novo* early PD patients, whereas we investigated patients with higher Hoehn and Yahr stage and greater motor disability at the Unified PD Rating Scale-motor section. It could be of interest to evaluate in follow-up studies how the serotonergic transmission damage may influence PD motor symptoms along with the progression of the disease. Concurrently, the reliability of the proposed difference in raphe 5-HT transporter availability found by Qamhawi and coworkers in early PD patients should be also analyzed in more advanced PD patients.

Remarkably, Qamhawi and coauthors documented that raphe 5-HT transporter availability did not correlate with PD non-motor symptoms, such as fatigue, depression, and sleep-wake cycle disturbances. In agreement with this finding, we did not identify mutual interplays linking CSF 5-HT and 5-HIAA concentrations to depression, apathy, and sleep disturbances. Although our report sustained Qamhawi and coworkers results, literature proposes controversial data regarding the involvement of serotonergic pathways breakage in the pathophysiology of PD non-motor symptoms (11), thus requiring further investigations.

All considering, we suppose that the reduced 5-HT transporter availability, as shown by Qamhawi and collegues, and the 5-HT synthesis, metabolism, and turnover impairment, resulting in reduced CSF levels of 5-HT and 5-HIAA (8) may both contribute to the serotonergic transmission dysfunction evident in PD.

Hence, serotonin circuitry inefficiency could represent the fitting partner of the dopaminergic deficit in PD.

## Author Contributions

CL: manuscript preparation; MP: manuscript preparation and critical review; EO: references suggestion; NM: critical review; AS: critical review.

## Conflict of Interest Statement

The authors declare that the research was conducted in the absence of any commercial or financial relationships that could be construed as a potential conflict of interest.
